# Climate change and indoor biological exposures: a hidden risk to immune health

**DOI:** 10.3389/fpubh.2025.1597881

**Published:** 2025-08-07

**Authors:** Hesham Amin, Randi J. Bertelsen

**Affiliations:** Department of Clinical Science, University of Bergen, Bergen, Norway

**Keywords:** climate change, microbial diversity, immune health, allergens and pathogens, public health impacts, ventilation and building design

## Introduction

**Figure 1:**
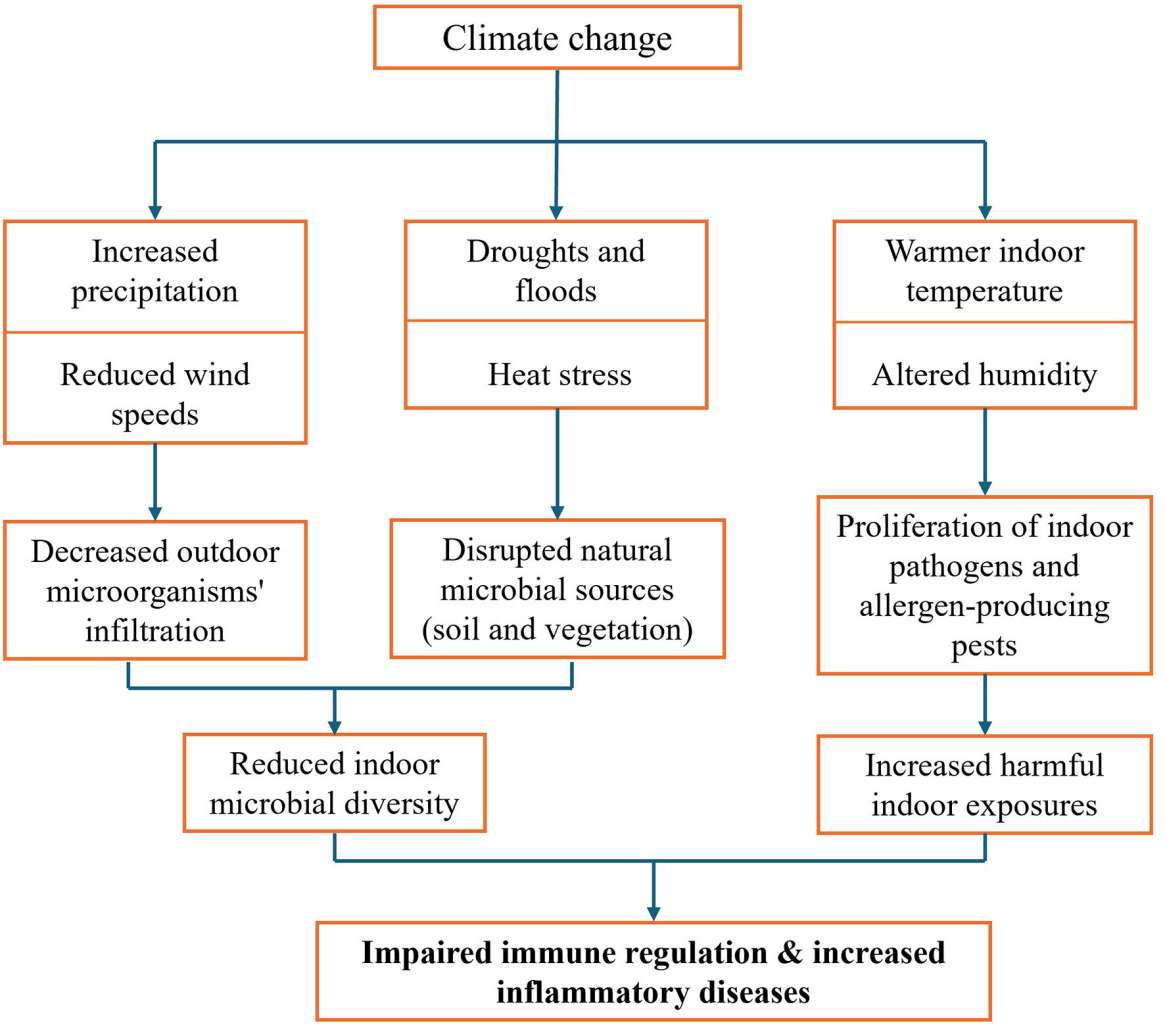
How climate change might alter indoor microbial exposures and immune health.

The impacts of climate change on ecosystems and weather patterns are widely recognized (1). However, its influence on indoor microbial exposure and biological exposure and subsequent effects on immune health remains underexplored. Climate-driven shifts, particularly increased precipitation and reduced wind speeds, limit the infiltration of outdoor microorganisms, decreasing indoor microbial diversity (2). This reduction may impair immune regulation and increase susceptibility to inflammatory diseases (3). In addition to limiting the infiltration of outdoor microbes, climate change is also disrupting the natural sources that contribute to indoor microbial diversity particularly soil and vegetation (4). These ecosystems are increasingly affected by droughts, floods and heat stress, which can diminish the availability of beneficial microbes entering indoor spaces, especially in urban settings. At the same time, warmer and more stable indoor climates common in modern energy-efficient buildings create favorable conditions for allergen-producing pests such as the long-tailed silverfish (5). Rising temperatures and altered humidity also support the proliferation of indoor pathogens and intensify exposure to environmental allergens (6, 7). Collectively, these shifts expand the ways in which climate change may reduce beneficial microbial diversity, increase harmful exposures, and disrupt immune system regulation within indoor environments which could have direct and widespread public health implications. This opinion piece explores how climate change alters indoor microbial and biological exposures through a web of interconnected pathways as illustrated in Figure 1.

## Microbial diversity, immune health, and climate-driven infiltration

Indoor environments host diverse microbial communities, including bacteria, fungi, and viruses, which interact closely with the human immune system. In this context, “microbial diversity” refers primarily to the richness of microbial species, especially those originating from outdoor environments like soil and vegetation. Exposure to these microorganisms trains the immune system to distinguish between harmful and harmless agents, supporting balanced immune responses (8, 9). The biodiversity hypothesis supports this concept, suggesting that reduced microbial exposure particularly early in life disrupts immune tolerance (3). Several studies support this connection. For instance, research comparing populations in Russian Karelia and Finnish Karelia found lower rates of asthma and allergies in Russian regions with higher indoor and environmental microbial diversity (10). Similarly in US, children raised in Amish communities, with high exposure to farm microbes had significantly lower asthma prevalence compared to genetically similar Hutterite children raised in industrialized environments (11).

Outdoor environments are the primary source of indoor microbial diversity, and climate-related meteorological conditions play a central role in determining microbial transport indoors (12). Wind speed and precipitation are particularly important while wind facilitate the transfer of airborne bacteria indoors, precipitation removes them through wet deposition (13). A Study in Nordic cities highlight this relationship where cities like Aarhus and Tartu, with higher wind speeds and lower rainfall, exhibit greater indoor bacterial diversity, whereas rainier cities like Bergen show reduced microbial infiltration (2). Additionally, modern sealed, energy-efficient buildings further restrict air exchange, compounding the effects of adverse weather patterns and limiting beneficial microbial influx. While design interventions such as green walls and improved ventilation are being explored (14, 15), the essential role of outdoor microbial influx driven by meteorological conditions remains underappreciated in public health strategies. However, the implementation of such interventions may face practical challenges, including cost, maintenance requirements especially in older buildings.

## Environmental microbiome disruption: soil and vegetation as climate-sensitive microbial sources

Soil and vegetation are key natural sources of airborne microbial diversity that influence indoor environments (16). However, climate-driven changes such as droughts, floods, rising temperatures, and altered precipitation patterns are reshaping these ecosystems, with downstream impacts on microbial exposure indoors. Soil, as a major microbial reservoir, contributes airborne microorganisms via dust and particulate matter (16, 17). Climate-induced declines in soil microbial richness and functional diversity (18) may reduce the availability of beneficial microbes entering homes. This effect is particularly concerning in urban settings, where direct contact with soil is already limited. A decrease in soil-derived microbial input may disturb the indoor microbial balance, restricting early-life exposure to environmental microbes that is vital for immune development and tolerance.

Similarly, vegetation including urban greenery and crops hosts diverse microbial communities on leaf surfaces, known as the phyllosphere (19). These microbes are released into the air and contribute to human microbial exposure (16). Yet, climate-related stressors can reduce microbial diversity on plants or promote the growth of opportunistic pathogens (20). Shifts in plant-associated microbial communities may, therefore alter the composition of airborne microbiota entering indoor spaces, with potential consequences for respiratory health and immune function. Recent global-scale studies support this link. For example, Chen et al. (22) found that climate type, vegetation cover, soil characteristics, and precipitation patterns are strong predictors of microbial community composition across regions. Moreover, a global study by Delgado et al. (21) across five continents found that soil microbial diversity particularly of bacteria, fungi, and protists—is strongly shaped by climate. These findings reinforce the idea that climate-driven ecological changes can significantly reshape the environmental microbiomes that influence indoor microbial exposure (22).

These disruptions to soil and vegetation-associated microbiomes reveal a largely underappreciated pathway by which climate change may influence the microbial composition of indoor environments. Preserving the microbial diversity of natural ecosystems is not only vital for environmental health but may also have important downstream benefits for human immune function, particularly in urban settings with limited exposure to nature.

## Pathogenic microorganism and allergen shifts: immune overactivation in a changing climate

Climate change is reshaping the distribution and prevalence of both beneficial and harmful microorganisms. Warmer temperatures, increased humidity, and altered precipitation patterns create favorable conditions for the growth of opportunistic indoor pathogens, such as molds, bacteria, and viruses (7). For example, prolonged dampness in poorly ventilated buildings can promote fungal growth including *Aspergillus* and *Penicillium* which are known to trigger immune responses and respiratory symptoms (23). Continuous exposure to such microorganisms may contribute to chronic low-grade inflammation and disrupt immune tolerance, increasing susceptibility to asthma, allergies, and autoimmune conditions (24). Particularly in urban environments with already reduced microbial diversity.

At the same time, climate change is expected to intensify indoor allergen exposures. Elevated atmospheric CO_2_ levels and rising global temperatures have been linked to increased pollen production and longer allergen seasons (25, 26). These allergens can infiltrate indoor spaces, especially in buildings lacking adequate filtration or ventilation, compounding the burden of environmental triggers on respiratory and immune health. While air filtration systems are often recommended to improve indoor air quality, their real-world effectiveness in reducing inflammation and allergic disease remains insufficiently studied.

Together, the rise in pathogenic and allergenic exposures underscores the need to better understand how climate-driven changes shape indoor biological risks. Developing robust, evidence-based strategies to address these interconnected exposures is critical for safeguarding immune health in a warmer and increasingly urbanized world.

## Indoor ecology, climate adaptation, and emerging allergen risks

As climate change accelerates global warming and alters humidity and precipitation patterns, indoor environments are also undergoing transformation. In response to energy demands, modern buildings are increasingly constructed with tightly sealed structures and controlled indoor climates to optimize thermal performance (27). However, inadequate ventilation systems or lack of maintenance and knowledge regarding modern climate control systems can unintentionally favor the establishment of indoor biological agents, including pests and opportunistic pathogens. But even well-balanced modern climate systems may open niches to new pests to establish themselves. A prominent example is the long-tailed silverfish (*Ctenolepisma longicaudata*), a resilient insect that has become increasingly widespread in newer buildings across Europe. First reported in Norway and the UK about a decade ago (5), and recent reviews have documented a sharp rise in its occurrence, especially in dwellings constructed within the past 15 years (28). Its growing presence appears to be linked to changes in indoor environmental conditions. In particular, consistently warm and dry indoor air common in climate-adapted homes during colder months may facilitate its survival and spread, even in regions where it was previously uncommon (29). The health implications of this trend are noteworthy. The long-tailed silverfish carries tropomyosin, a potent allergen also found in house dust mites and other arthropods (30).

This illustrates how climate-driven changes in both environment and architecture can shape indoor biological exposures in unforeseen ways. Expanding our understanding of how climate adaptation affects pest ecology, microbial communities, and allergen profiles will be essential for safeguarding immune health in an increasingly climate-modified indoor environment.

## Discussion

Climate change is altering atmospheric dynamics, intensifying rainfall and reducing wind speeds. Warmer temperatures increase atmospheric moisture thereby amplifying precipitation (31), while the Arctic warms twice as fast as the global average, reducing the temperature difference between the poles and the equator. This weakening gradient is expected to lower average wind speeds (32).

These meteorological changes, alongside rising global temperatures and humidity, influence the diversity and composition of microbes entering indoor spaces (2). Reduced outdoor-to-indoor microbial exchange, particularly in tightly sealed urban buildings, limits human exposure to beneficial microorganisms (15). In turn, this may impair immune system maturation and tolerance, increasing the risk of inflammatory diseases such as allergies, asthma, and autoimmune conditions (3). Despite its relevance, this subtle consequence of climate change is largely underrecognized in public health discussions.

Beyond meteorological shifts, climate-driven disruptions in the broader environment further compound these effects. Soil and vegetation, two critical sources of airborne microbial diversity are increasingly affected by droughts, floods, heat stress, and changing precipitation patterns (4, 18, 20). These stressors reduce the richness and stability of microbial communities in both soil and plant surfaces, ultimately lowering the diversity of environmental microbes that infiltrate indoor environments (18–20). This issue is particularly relevant in urban areas, where direct contact with natural ecosystems is already limited (16). Reduced exposure to diverse soil- and plant-associated microbes, particularly during early life, may interfere with immune system training and contribute to hypersensitivity and allergic disease (9).

In addition to reducing beneficial microbial exposures, climate change shifts the indoor biological landscape in more harmful directions. Earlier sections of this paper have described how warming, humidity, and extended growing seasons support the growth of opportunistic pathogens and prolong allergen exposure indoors. These trends, when combined with declining microbial diversity, may result in a dual burden greater exposure to irritants and reduced microbial signals necessary for immune regulation.

Importantly, these biological risks are not uniform and likely to vary across regions. Differences in local climate, vegetation types, soil composition, and ventilation practices will shape how climate change impacts indoor microbial exposure. For instance, the types of allergenic species or microbial taxa affected may differ between temperate and tropical zones, or between rural and highly urbanized settings. While the underlying mechanisms are globally relevant, the outcomes will be context-specific and should be studied with attention to regional differences.

Indoor architecture also plays a role. Tightly sealed modern buildings with poor ventilation or moisture control, may unintentionally create ecological niches that favor emerging biological threats such as the long-tailed silverfish. This species has been increasingly reported in European homes, particularly in newer buildings (28). Its allergenic potential due to the presence of tropomyosin and its ability to move between housing units make it difficult to eliminate once established, raising concerns about long-term health impacts (28, 30). Notably, DNA sequencing of household dust, commonly used in microbiome research, can detect not only microbial communities but also genetic material from indoor pests (33). This underscores the interconnected nature of microbial and allergen exposures in modern indoor environments.

Addressing these interconnected challenges requires interdisciplinary approach involving microbiologists, environmental scientists, urban planners, and public health professionals. Public health strategies should evolve to preserve beneficial microbial diversity while mitigating harmful exposures. This includes integrating green infrastructure, enhancing ventilation systems, and designing buildings that consider the ecological dynamics of indoor air.

Further research is needed to quantify the long-term effects of reduced microbial exposure and inform strategies that preserve microbial diversity, safeguarding public health in an increasingly urbanized, warming world.

Altogether, this opinion highlights that climate change alters indoor biological exposures through a web of interconnected pathways—meteorological shifts, environmental microbiome disruption, indoor architectural adaptations, and allergen dynamics. Understanding and addressing these emerging risks will be essential to creating resilient, health-supportive indoor environments in the face of accelerating climate change.
